# Reducing energy consumption in musculoskeletal MRI using shorter scan protocols, optimized magnet cooling patterns, and deep learning sequences

**DOI:** 10.1007/s00330-024-11056-0

**Published:** 2024-09-07

**Authors:** Saif Afat, Julian Wohlers, Judith Herrmann, Andreas S. Brendlin, Sebastian Gassenmaier, Haidara Almansour, Sebastian Werner, Jan M. Brendel, Alexander Mika, Christoph Scherieble, Mike Notohamiprodjo, Sergios Gatidis, Konstantin Nikolaou, Thomas Küstner

**Affiliations:** 1 Department of Radiology, Tuebingen University Hospital, University of Tuebingen, Tuebingen, Germany; 2 Department of Magnetic Resonance Product Management, Siemens Healthineers, Erlangen, Germany; 3 Radiologische, Strahlentherapeutische und Nuklearmedizinische Partnerschaftsgesellschaft Muenchen, DIE RADIOLOGIE, Munich, Germany

**Keywords:** Magnetic resonance imaging, Sustainability, Deep learning, Energy consumption, Accelerated imaging

## Abstract

**Objectives:**

The unprecedented surge in energy costs in Europe, coupled with the significant energy consumption of MRI scanners in radiology departments, necessitates exploring strategies to optimize energy usage without compromising efficiency or image quality. This study investigates MR energy consumption and identifies strategies for improving energy efficiency, focusing on musculoskeletal MRI. We assess the potential savings achievable through (1) optimizing protocols, (2) incorporating deep learning (DL) accelerated acquisitions, and (3) optimizing the cooling system.

**Materials and methods:**

Energy consumption measurements were performed on two MRI scanners (1.5-T Aera, 1.5-T Sola) in practices in Munich, Germany, between December 2022 and March 2023. Three levels of energy reduction measures were implemented and compared to the baseline. Wilcoxon signed-rank test with Bonferroni correction was conducted to evaluate the impact of sequence scan times and energy consumption.

**Results:**

Our findings showed significant energy savings by optimizing protocol settings and implementing DL technologies. Across all body regions, the average reduction in energy consumption was 72% with DL and 31% with economic protocols, accompanied by time reductions of 71% (DL) and 18% (economic protocols) compared to baseline. Optimizing the cooling system during the non-scanning time showed a 30% lower energy consumption.

**Conclusion:**

Implementing energy-saving strategies, including economic protocols, DL accelerated sequences, and optimized magnet cooling, can significantly reduce energy consumption in MRI scanners. Radiology departments and practices should consider adopting these strategies to improve energy efficiency and reduce costs.

**Clinical relevance statement:**

MRI scanner energy consumption can be substantially reduced by incorporating protocol optimization, DL accelerated acquisition, and optimized magnetic cooling into daily practice, thereby cutting costs and environmental impact.

**Key Points:**

- *Optimization of protocol settings reduced energy consumption by 31% and imaging time by 18%*.
- *DL technologies led to a 72% reduction in energy consumption of and a 71% reduction in time, compared to the standard MRI protocol*.
- *During non-scanning times, activating Eco power mode (EPM) resulted in a 30% reduction in energy consumption, saving 4881* *€ ($5287) per scanner annually*.

## Introduction

The energy cost in the Eurozone has experienced an unprecedented surge, with the price more than doubling between December 2020 and December 2021 [1]. This rising energy price in Europe has spotlighted radiology departments and practices. Accounting for at least 4.2% of a hospital’s total energy consumption, the radiology department is also a significant contributor to greenhouse gas emissions [2–7]. As the demand for medical devices like magnetic resonance imaging (MRI) and computed tomography (CT) scanners grows, their energy consumption has become a pressing concern for radiology departments and practices. These devices are the primary energy consumers in such facilities, and their efficient use has become a top priority. Within the radiology department, each MRI scanner consumes on average more than 100 MWh per year, higher than any other equipment [8, 9]. Several recent studies investigated the energy consumption of radiology departments and emphasized initiatives aimed at improving the energy efficiency of MRI scanners [9–14].

In principle, shortening the acquisition times, i.e., shorter scanner operation, will directly lead to reduced energy consumption. Nevertheless, the same diagnostic capability should be maintained. Optimizing protocols, implementing new standby modes, and incorporating deep learning (DL) sequences are, thus, potential solutions to reduce energy consumption. Due to the constant operation of the cold head cooling system (to keep the superconducting state of the magnet), MRI scanners consume between 31% and 38% of their total annual energy usage during the scanner-off system state [9]. Examining the individual components of an MRI, it becomes apparent that the magnet cooling and gradient are the primary drivers of energy consumption. Based on the data sheets of Siemens Healthineers and combined with conducted measurements, magnet cooling alone accounts for approximately 42% of total energy consumption [15]. Therefore, optimization of the cooling system and reduction of the active phases of the gradients are crucial to reducing the overall energy consumption of the MRI. Novel developments in accelerating the MRI acquisition by DL show promising results. DL can help to overcome tradeoffs regarding acquisition time, signal-to-noise ratio, and spatial and temporal resolutions. In MR image reconstruction, DL methods benefit from the vast amount of undersampled (i.e., sub-Nyquist sampled) or noisy data to reconstruct high-fidelity images. DL algorithms thereby either operate and integrate the undersampled raw data, i.e., data acquired from faster scans, to reconstruct high-quality outputs or they operate in the image domain to denoise the image either ad hoc or integrated into an iterative reconstruction algorithm [16–18]. These DL accelerated acquisitions can balance scan quality and acquisition times. In fact, they reduce the acquisition time for a specific MRI sequence without compromising the image quality [19–28].

This study aims to investigate MRI scanner energy consumption and to identify strategies for improving energy efficiency in radiology departments and practices, including protocol optimization, incorporation of DL accelerated acquisitions, and cooling system optimization.

## Material and methods

**Fig. 1:**
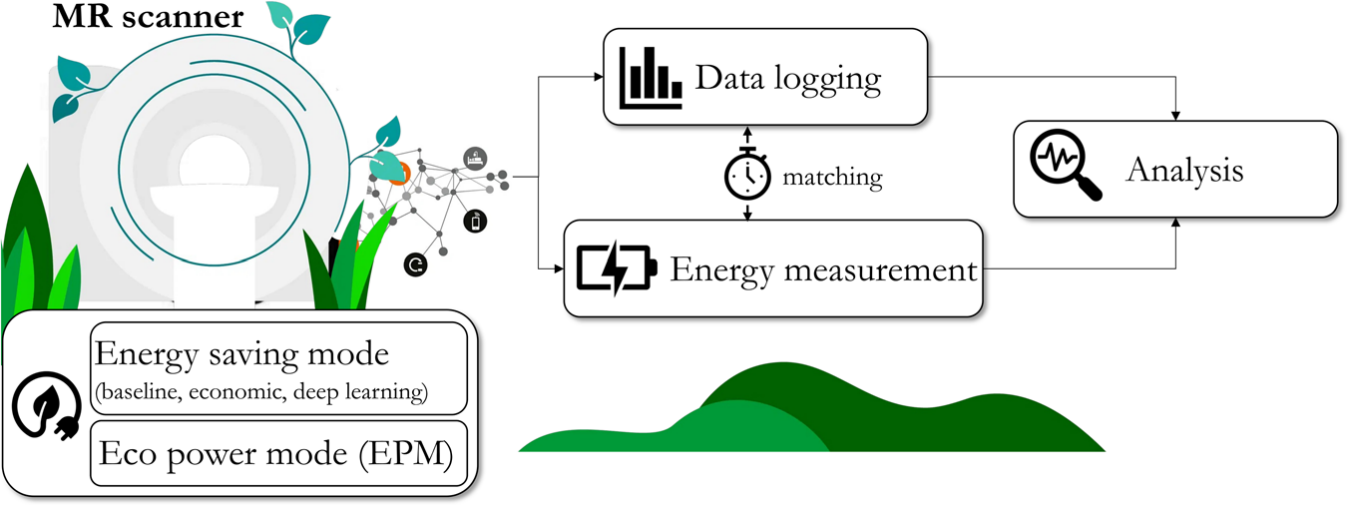
Overview of proposed energy reduction study. For the two MR scanners, the various energy-saving protocol modes—baseline (no modifications), economic (shortened sequences with conventional accelerations), and DL (DL-accelerated sequences)—were investigated and the respective energy consumption, scan times, and scanner idle times were measured and analyzed

The prospective measurement of total energy consumption of two 1.5-T MRI scanners (Siemens Aera and Sola, Siemens Healthineers) was intended to calculate annual usage. We aimed to reduce energy consumption by various measures available from the vendor (economic protocols and eco power mode (EPM)) and by novel DL accelerated acquisitions. The following two energy-saving modes can thus be differentiated: “economic” (protocols) with shortened scan protocols; “DL” with DL-accelerated sequences. We focused our investigations on musculoskeletal MRI. The workflow is depicted in Fig. 1.

### Energy consumption measurements

Measurements were performed between the 6th of December 2022 and the 20th of March 2023 at two registered private practices in the metropolitan area of Munich, Germany, each equipped with an MRI scanner: 1.5-T MRI (Magnetom Aera, Siemens Healthineers, software version VA30) at site A and a 1.5-T MRI (Magnetom Sola, software version VA50A, Siemens Healthineers) at site B. Normal operation hours of the practices are on average between 8 h and 12 h per day. Over the course of four months, 2252 (Aera)/3214 (Sola) musculoskeletal examinations were performed from which 262 (Aera)/372 (Sola) were scanned with economic protocols (as detailed below) and 186 (Sola) DL examinations were conducted. Protocol decisions were made on a case-by-case basis, depending on available scan time, patient compliance, and consent.

Power consumption was measured non-invasively with a UMG 512 power quality analyzer from Janitza over the course of four months. Measurements of the voltage [*V*], current [*A*], effective power [*W*], and reactive power [VAR] were performed in three phases at a 20 kHz sampling rate on the main power lines to the MR scanner with logged timestamps. Time was synchronized to the MR scanner via a network time protocol server. The measurements were then analyzed over a 10-min interval. The measured energy consumption (integral of power consumption over time [Wh]) includes the operation of the MR scanner, magnet cooling, and the reconstruction server, but not the external cooling components (chiller).

Two levels of energy reduction measures were implemented and studied at the protocol level. First, the so-called economic protocols and second DL accelerated sequences were compared to the baseline (i.e., no energy reduction measures). The economic protocols were available on both MRI scanners studied (1.5-T Aera and 1.5-T Sola), while the DL accelerated sequences were only available on the 1.5-T Sola scanner. The economic protocols include shortened sequence protocol trees with slightly lower imaging resolution and increased parallel imaging acceleration factors (conventional imaging acceleration). In both scanners, the energy consumption of the magnet cooling accounts for a major share of energy. It has been further optimized by switching off the helium pump during off-cycles and reactivating it based on temperature and pressure values. This measure is called EPM, which was performed independently of the other techniques and was intended to contribute to an additional power reduction. DL accelerated sequences (DeepResolve, Siemens Healthineers) replaced the conventional sequences where available, enabling higher acceleration factors of the regular parallel imaging sub-Nyquist sampling, i.e., yielding shorter scan times. The stronger aliasing-artifact affected images (compared to economic mode or baseline) were reconstructed on the reconstruction server at the scanner (energy consumption included in the measurement) by a vendor-provided DL reconstruction network called DeepResolve Boost [29]. While computational reconstruction demand for the DL accelerated sequences is increased compared to economic mode or baseline, the shorter acquisition time, and hence reduced energy consumption by less played-out RF pulses and gradients, is expected to compensate for this, Fig. 1.

A musculoskeletal MRI was performed on the hip, knee, spine, and shoulder. The following protocols were performed in a conventional program tree for these anatomical regions. In the hip: localizer, T1 coronal, proton density fat-saturated coronal, transversal, and sagittal; in the knee: localizer, T1 sagittal, proton density fat-saturated sagittal, transversal, and coronal; in the shoulder: localizer, T2 sagittal, T1 sagittal, T2 fat-saturated coronal (for DL: short-tau inversion recovery coronal), and T2 transversal; and in the spine: localizer, proton density fat-saturated transversal, T1 sagittal, T2 fat-saturated sagittal, and proton density fat-saturated coronal. Selected imaging parameters for the hip, knee, spine, and shoulder are specified in Tables 1–4. All images were inspected by experienced readers (S.A., J.H., S.G., and M.N.).

**Table 1:** MRI parameters of the protocols in the hip for baseline, and the investigated energy-saving modes: economic and DL

	Baseline	Economic	DL
T1 COR nativ			
TE	12 ms	12 ms	10 ms
TR	713 ms	713 ms	456 ms
Acceleration factor	2	4	6
Slices	32	32	32
Voxel size	0.72 × 1.0 × 3.0 mm	0.72 × 1.0 × 3.0 mm	0.47 × 0.46 × 3.0 mm
TA	2:11 min:s	1:09 min:s	0:50 min:s
FoV	300 × 300 mm	300 × 300 mm	300 × 300 mm
PD FS COR nativ			
TE	43 ms	43 ms	42 ms
TR	3230 ms	3230 ms	2610 ms
Acceleration factor	2	4	6
Slices	24	24	24
Voxel size	0.71 × 0.89 × 3.0 mm	0.71 × 0.89 × 3.0 mm	0.36 × 0.4 × 3.0 mm
TA	3:10 min:s	1:35 min:s	1:23 min:s
FoV	240 × 240 mm	240 × 240 mm	240 × 240 mm
PD FS TRA nativ			
TE	43 ms	43 ms	41 ms
TR	3520 ms	3520 ms	2500 ms
Acceleration factor	2	4	6
Slices	26	26	28
Voxel size	0.69 × 0.89 × 3.0 mm	0.69 × 0.89 × 3.0 mm	0.375 × 0.44 × 3.0 mm
TA	3:06 min:s	1:33 min:s	1:17 min:s
FoV	240 × 240 mm	240 × 240 mm	240 × 240 mm
PD FS SAG nativ			
TE	44 ms	44 ms	42 ms
TR	2920 ms	2920 ms	3110 ms
Acceleration factor	2	4	6
Slices	36	36	36
Voxel size	0.75 × 0.94 × 3.0 mm	0.75 × 0.94 × 3.0 mm	0.375 × 0.44 × 3.0 mm
TA	2:46 min:s	1:23 min:s	1:20 min:s
FoV	240 × 240 mm	240 × 240 mm	240 × 240 mm

**Table 2:** MRI parameters of the protocols in the knee for baseline, and the investigated energy-saving modes: economic and DL

	Baseline	Economic	DL
T1 SAG nativ			
TE	11 ms	13 ms	11 ms
TR	690 ms	632 ms	539 ms
Acceleration factor	2	4	8
Slices	30	32	30
Voxel size	0.4 × 0.4 × 3.0 mm	0.4 × 0.4 × 3.0 mm	0.2 × 0.2 × 3.0 mm
TA	2:28 min:s	1:11 min:s	0:49 min:s
FoV	160 × 160 mm	160 × 160 mm	160 × 160 mm
PD FS SAG nativ			
TE	32 ms	41 ms	43 ms
TR	3400 ms	2830 ms	2500 ms
Acceleration factor	2	4	6
Slices	32	32	30
Voxel size	0.4 × 0.4 × 3.0 mm	0.5 × 0.5 × 3.0 mm	0.2 × 0.2 × 3.0 mm
TA	2:28 min:s	1:38 min:s	1:11 min:s
FoV	160 × 160 mm	160 × 160 mm	160 × 160 mm
PD FS COR nativ			
TE	46 ms	45 ms	43 ms
TR	3370 ms	3730 ms	2500 ms
Acceleration factor	2	2	6
Slices	27	25	28
Voxel size	0.4 × 0.4 × 3.0 mm	0.5 × 0.5 × 3.0 mm	0.2 × 0.2 × 3.0 mm
TA	2:20 min:s	1:59 min:s	1:01 min:s
FoV	160 × 160 mm	160 × 160 mm	160 × 160 mm
PD FS TRA nativ			
TE	39 ms	40 ms	42 ms
TR	3330 ms	3060 ms	2550 ms
Acceleration factor	2	4	6
Slices	30	34	34
Voxel size	0.5 × 0.5 × 3.0 mm	0.5 × 0.5 × 3.0 mm	0.3 × 0.3 × 3.0 mm
TA	2:05 min:s	1:52 min:s	1:08 min:s
FoV	160 × 160 mm	160 × 160 mm	160 × 160 mm

**Table 3:** MRI parameters of the protocols in the spine for baseline, and the investigated energy-saving modes: economic and DL

	Baseline	Economic	DL
T2 SAG nativ			
TE	108 ms	108 ms	104 ms
TR	4500 ms	5910 ms	4700 m
Acceleration factor	2	4	8
Slices	16	19	19
Voxel size	0.78 × 0.82 × 4.0 mm	0.78 × 0.82 × 3.0 mm	0.36 × 0.4 × 3.0 mm
TA	2:01 min:s	1:46 min:s	1:10 min:s
FoV	300 × 300 mm	300 × 300 mm	300 × 300 mm
T1 SAG nativ			
TE	9.4 ms	12 ms	7.8 ms
TR	672 ms	497 ms	517 ms
Acceleration factor	2	4	8
Slices	16	19	19
Voxel size	0.75 × 1.0 × 4.0 mm	0.9 × 0.95 × 3.0 mm	0.39 × 0.43 × 3.0 mm
TA	1:53 min:s	1:40 min:s	1:10 min:s
FoV	300 × 300 mm	300 × 300 mm	300 × 300 mm
STIR COR nativ/T2 FS COR nativ			
TE	104 ms	84 ms	56 ms
TR	4640 ms	4630 ms	4590 ms
TI	N/A	N/A	160
Acceleration factor	2	4	8
Slices	19	19	30
Voxel size	0.8 × 0.94 × 4.0 mm	0.95 × 0.88 × 3.0 mm	0.66 × 0.58 × 3.0 mm
TA	1:28 min:s	2:32 min:s	1:36 min:s
FoV	320 × 320 mm	380 × 380 mm	380 × 380 mm
T2 TRA nativ			
TE	111 ms	100 ms	99 ms
TR	4220 ms	4470 ms	3570 ms
Acceleration factor	2	4	6
Slices	36	38	38
Voxel size	0.63 × 0.83 × 4 mm	0.69 × 0.95 × 3.0 mm	0.34 × 0.39 × 3.0 mm
TA	2:50 min:s	2:50 min:s	1:26 min:s
FoV	200 × 200 mm	220 × 220 mm	200 × 200 mm

**Table 4:** MRI parameters of the protocols in the shoulder for baseline, and the investigated energy-saving modes: economic and DL

	Baseline	Economic	DL
PD FS TRA nativ			
TE	44 ms	45 ms	39 ms
TR	3180 ms	3860 ms	3340 ms
Acceleration factor	2	4	8
Slices	25	25	26
Voxel size	0.42 × 0.52 × 3.0 mm	0.45 × 0.51 × 3.0 mm	0.25 × 0.31 × 3.0 mm
TA	3:36 min:s	2:38 min:s	1:23 min:s
FoV	160 × 160 mm	160 × 160 mm	160 × 160 mm
T1 SAG nativ			
TE	12 ms	14 ms	13 ms
TR	683 ms	692 ms	504 ms
Acceleration factor	2	4	8
Slices	26	24	24
Voxel size	0.36 × 0.44 × 3.0 mm	0.32 × 0.4 × 3.0 mm	0.16 × 0.2 × 3.0 mm
TA	1:21 min:s	1:16 min:s	1:08 min:s
FoV	160 × 60 mm	160 × 160 mm	160 × 160 mm
T2 FS SAG nativ			
TE	76 ms	80 ms	97 ms
TR	5280 ms	5140 ms	5780 ms
Acceleration factor	2	3	8
Slices	22	22	22
Voxel size	0.41 × 0.52 × 3.0 mm	0.45 × 0.57 × 3.0 mm	0.23 × 0.29 × 3.0 mm
TA	2:49 min:s	2:49 min:s	1:09 min:s
FoV	160 × 160 mm	160 × 160 mm	160 × 160 mm
PD FS COR nativ			
TE	40 ms	46 ms	49 ms
TR	3180 ms	2460 ms	3860 ms
Acceleration factor	2	3	8
Slices	22	22	22
Voxel size	0.42 × 0.52 × 3.0 mm	0.42 × 0.46 × 3.0 mm	0.2 × 0.23 × 3.0 mm
TA	2:42 min:s	2:20 min:s	1:44 min:s
FoV	160 × 160 mm	160 × 160 mm	160 × 160 mm

In addition to measuring energy consumption, all activities on the MR scanner were logged and analyzed by a vendor-provided platform (Siemens Fleet Cockpit). Logged activities include a timestamp and protocol, the start and end time of a patient examination, sequences, the start and end time of measurements, and certain user behaviors such as table control operation. Energy consumption measurements were matched to the MR scanner activity logs using the timestamps. The energy measurements were thus segmented into different states of the scanner: active measurements, idle times, and system off times.

### Statistical analysis

Statistical analysis was conducted in R (version 4.2.3) and Python (version 3.9). We evaluated the average, median, and standard deviations of the sequence scan times and energy consumption for the different energy-optimized protocols (baseline, economic, and DL), including optimized magnet cooling. Additional filtering was conducted based on the opening and closing times of the radiology practice. Outliers were defined as larger/smaller than 1.5 times the interquartile range. Statistical significance was performed with a Wilcoxon signed-rank test for a null hypothesis with a significance level of ɑ = 0.05 and Bonferroni correction. For calculated cost savings, we assumed an energy price of 0.50 €/kWh [30] which is the average energy price in Germany during the investigated timeframe.

## Results

### Scan time measurements

**Fig. 2:**
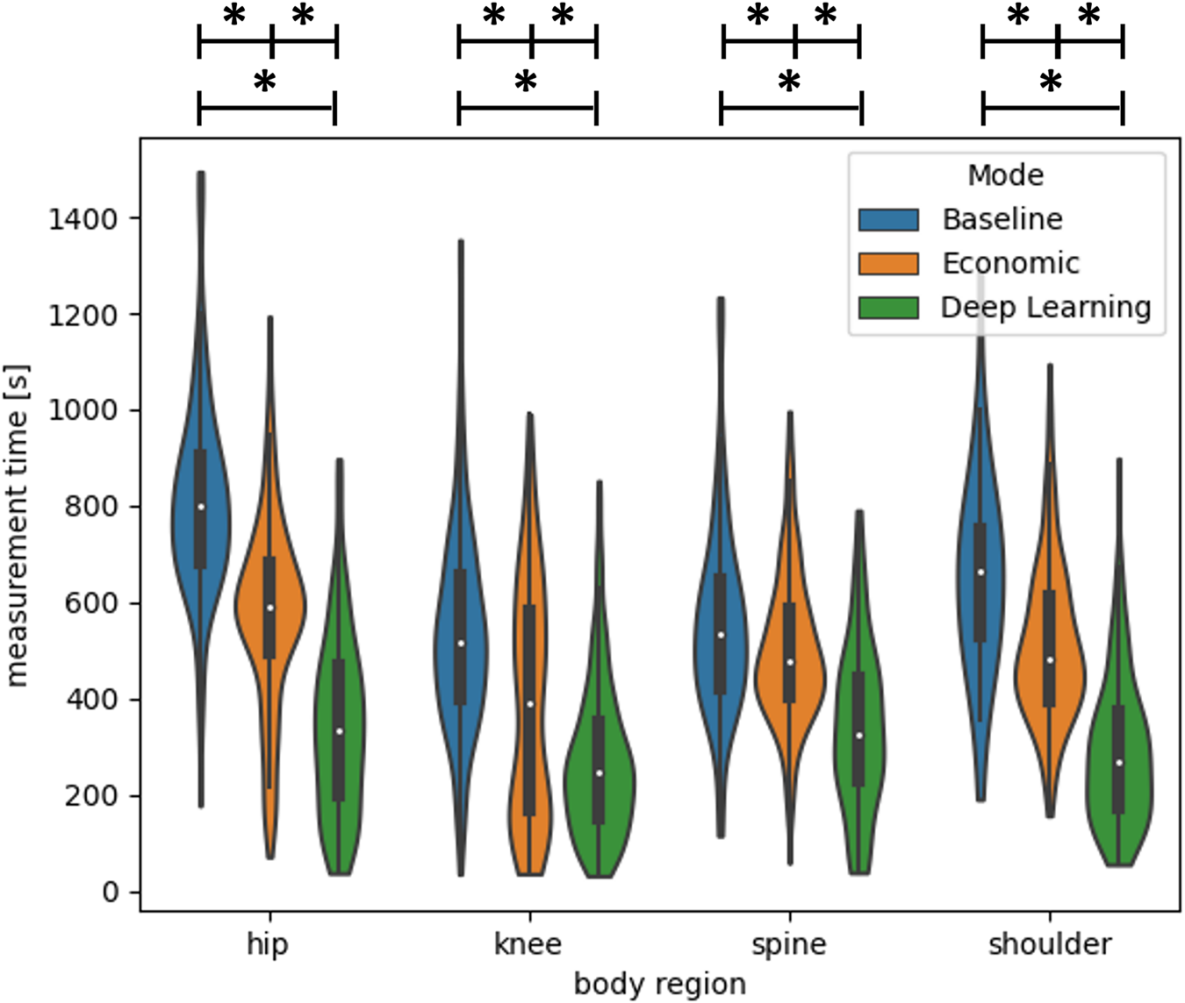
Acquisition times for each body region (hip, knee, spine, and shoulder) of the musculoskeletal protocols. Energy consumption modes are depicted: baseline (no modifications), economic (shortened sequences), and DL (DL-accelerated sequences). Data distribution, mean (white dot), interquartile range (solid black vertical box), and standard deviation (thin black vertical bars) are shown. Statistical significance with respect to a *p*-value of 0.05 is indicated by the star symbol

Among other factors, the reduction of scan time significantly contributes to energy savings, although several additional variables (sequence, application, targeted body region, …) also impact the total consumed energy. Figure 2 depicts the saved measurement time in each body region for the proposed energy-saving modes in comparison to the unmodified baseline. The average ± standard deviation recorded scan time for the complete protocol is reported for baseline, economic, and DL in Table 5. On average, the energy-saving economic protocols resulted in scan time reductions of 34% (hip; 235 s, *p* < 0.001), 32% (knee; 148 s, *p* < 0.001), 11% (spine; 57 s, *p* < 0.01), and 25% (shoulder; 146 s, *p* < 0.01) compared to the baseline. DL protocols in turn resulted in an average scan time reduction of 81% (hip; 467 s, *p* < 0.001), 66% (knee; 268 s, *p* < 0.001), 49% (spine; 218 s, *p* < 0.001), and 77% (shoulder; 367 s, *p* < 0.001) over the baseline, and of a 50% (hip; 232 s, *p* < 0.001), 36% (knee; 120 s, *p* < 0.001), 38% (spine; 161 s, *p* < 0.001), and 55% (shoulder; 221 s, *p* < 0.001) over the economic protocols. Scan time was significantly reduced over the baseline in all examined body regions for economic and DL energy-saving modes. For all body regions, an average reduction of 71% (DL) and 18% (economic protocols) over baseline was achieved.

**Table 5:** Scan time and energy consumption of investigated protocols: baseline (B), economic protocols (E), and DL protocols

	Hip			Knee			Spine			Shoulder		
	B	E	DL	B	E	DL	B	E	DL	B	E	DL
Scan time [s]	812 ± 209	577 ± 197	345 ± 186	538 ± 214	390 ± 243	270 ± 159	556 ± 201	499 ± 154	338 ± 171	657 ± 215	512 ± 163	290 ± 153
Energy consumption [kWh]	10.2 ± 4.1	8.3 ± 3.7	4.9 ± 2.6	10.2 ± 4.3	6.3 ± 3.2	3.9 ± 2.0	11.7 ± 4.3	9.4 ± 3.9	5.9 ± 2.8	11.9 ± 4.3	8.9 ± 4.0	5.3 ± 2.8

### Energy consumption measurements

**Fig. 3:**
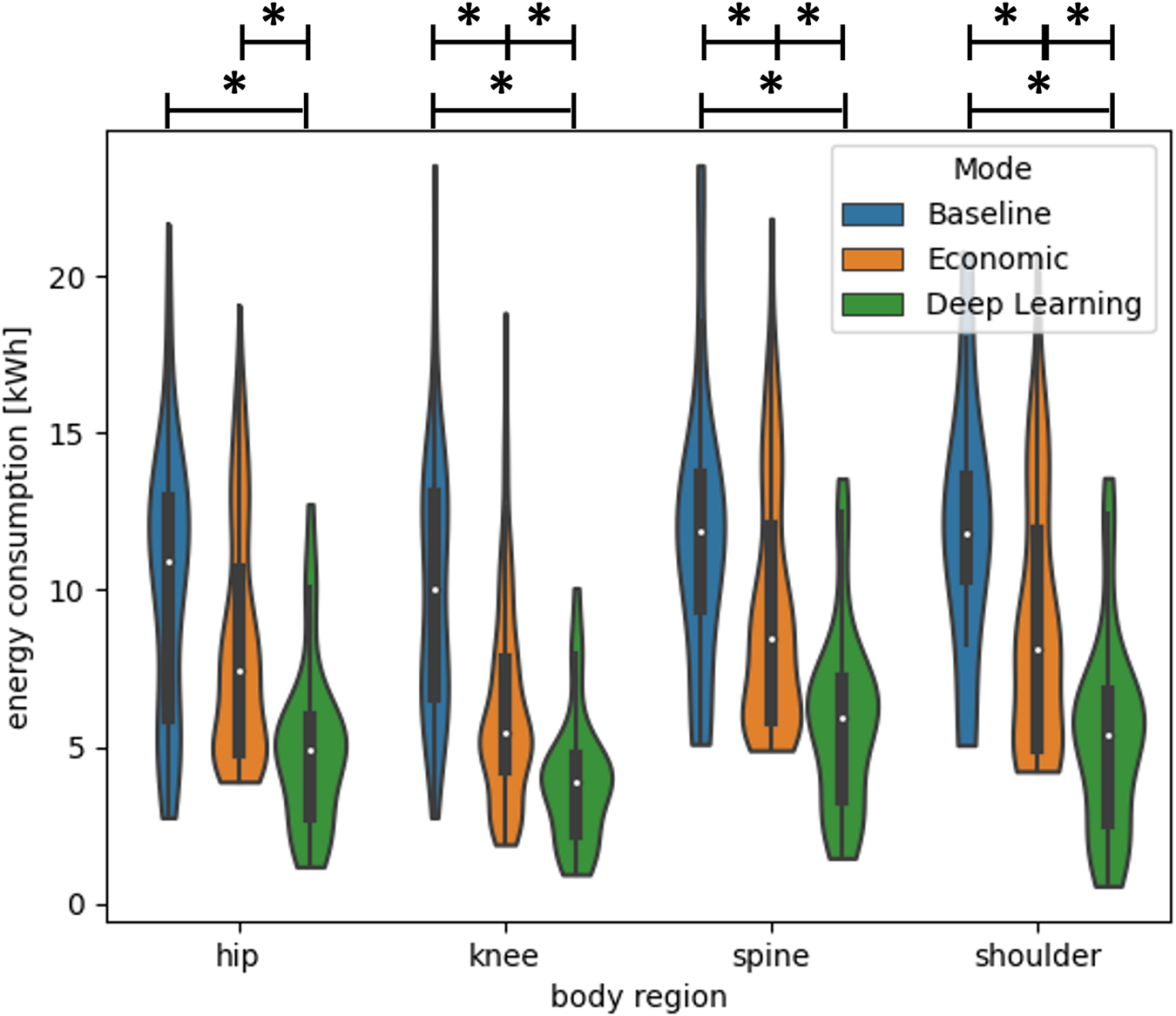
Energy consumption for each body region (hip, knee, spine, and shoulder) of the musculoskeletal protocols. Energy consumption modes are depicted: baseline (no modifications), economic (shortened sequences), and DL (DL-accelerated sequences). Data distribution, mean (white dot), interquartile range (solid black vertical box), and standard deviation (thin black vertical bars) are shown. Statistical significance with respect to a *p*-value of 0.05 is indicated by the star symbol

The associated energy consumption for each body region is shown in Fig. 3. The average ± standard deviation measured energy consumption for the complete protocol is reported for baseline, economic, and DL in Table 5. An average energy reduction of 21% (hip; 1.9 kWh, *p* = 0.31), 48% (knee; 4.0 kWh, *p* < 0.001), 22% (spine; 2.3 kWh, *p* < 0.01), and 28% (shoulder; 2.9 kWh, *p* < 0.01) was achieved with the economic protocols over baseline. In comparison to the baseline, the DL protocols provided an average energy consumption reduction of 69% (hip; 5.2 kWh, *p* < 0.001), 89% (knee; 6.3 kWh, *p* < 0.001), 66% (spine; 5.8 kWh, *p* < 0.001), and 76% (shoulder; 6.5 kWh, *p* < 0.001). The DL protocols achieved an additional energy reduction of 50% (hip; 3.3 kWh, *p* < 0.001), 45% (knee; 2.3 kWh, *p* < 0.001), 46% (spine; 3.5 kWh, *p* < 0.001), and 51% (shoulder; 3.6 kWh, *p* < 0.001) over the economic protocols. Energy consumption was significantly reduced from the baseline in all examined body regions for economic modes (except in the hip) and DL energy-saving modes. For all body regions, an average reduction of energy consumption of 72% (DL) or 31% (economic protocols) over baseline was achieved. Considering 250 working days with an average operation time of 8–12 h/day, 115 days without operation, and 0.5 €/kWh, 7032 € ($7617), or 5422 kg CO_2_ (385 g CO_2_/kWh in Germany) can be saved per scanner per year if DL protocols are applied.

### EPM

For energy reduction analysis of the independent EPM, the 10-min slots have been filtered for night times without procedures from 8:00 pm to 6:00 am. This, in turn, resulted in 2868 slots. Idle energy consumption without EPM was 1.33 ± 0.01 kWh (min: 1.32 kWh and max: 1.34 kWh), and with activated EPM was 1.02 ± 0.52 kWh (min: 0.14 kWh and max: 1.36 kWh). Thus, the average energy consumption during the non-scanning time is 30% lower if the EPM is activated. This is a significant reduction in energy consumption. Considering 250 working days with 10 h standby overnight, 115 days without operation, and 0.5 €/kWh this leads to yearly savings of 4881 € ($5287) per scanner per year at the investigated site. This optimization comes without compromises on image acquisition durations, image quality, or impact on scanner operations. Combining EPM with DL protocols can thus result in a yearly cost reduction of 11,913 € ($12,904) per scanner.

The log files also recorded the helium level of the MR scanner over time. The EPM, which switched the helium pump on/off between duty and non-duty cycles, did not result in any loss of helium over time. A constant helium level was recorded, and the helium pump was always automatically toggled on whenever temperature and pressure measurements demanded it. An optimized cooling cycle was hereby achieved.

### Qualitative image quality

**Fig. 4:**
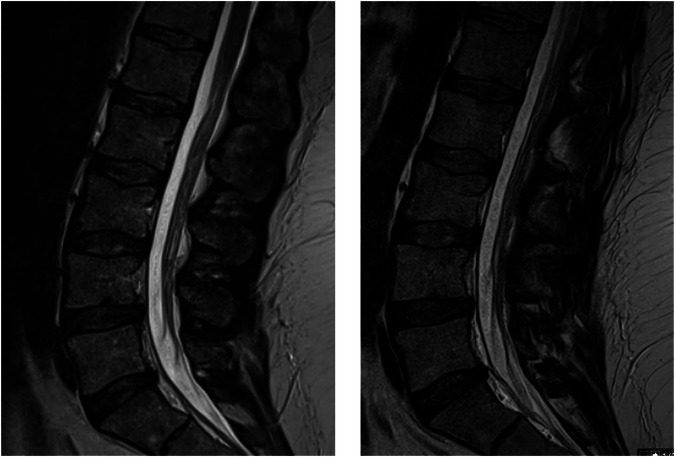
Example of standard spine T2 TSE (left) with an acquisition of 2:24 min and DL reconstructed (right) with an acquisition time of 1:13 min in sagittal orientation

**Fig. 5:**
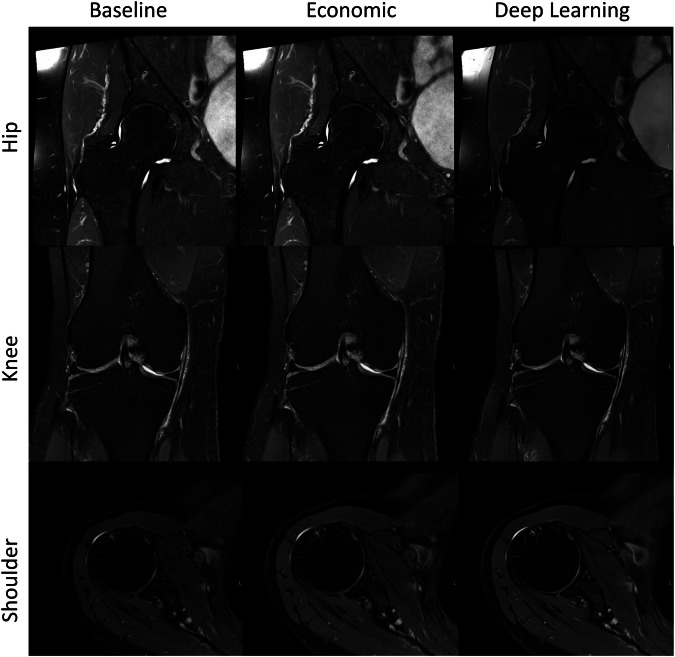
Example of the standard hip (top row), knee (middle row), and shoulder (bottom row) T2 TSE for baseline (left column), economic (shortened sequence) (middle column), and DL accelerated (right column) sequences. An acquisition time in hip/knee/shoulder for baseline of 3:21 min/3:08 min/3:01 min, for economic of 2:42 min/2:11 min/2:08 min, and for DL of 1:26 min/0:59 min/1:04 min were achieved

Representative image examples are displayed in Figs. 4 and 5, demonstrating comparable image quality between the accelerated acquisitions (economic, DL) and the conventional clinical baseline, however with reduced acquisition times. No relevant observable image quality differences were reported by the readers (S.A., J.H., S.G., and M.N.).

## Discussion

Our study was able to provide a comprehensive assessment of MRI scanner energy consumption and demonstrate how the application of certain techniques can contribute to significant energy savings. Through the optimization of protocol settings and the incorporation of DL, energy consumption was reduced by up to 56%, and imaging time decreased by up to 55%. Additionally, the use of EPM during idle times saved 30% in energy costs.

Heye et al revealed that MRI scanners consumed a significant amount of energy. They found that the aggregated energy consumption of three CT and four MRI scanners totaled 614,825 kWh in 2015, while adjunct cooling systems required an additional 492,624 kWh. The combined energy consumption of these imaging units and their cooling systems amounted to 4% of the hospital’s total annual energy consumption. This study provides an essential basis for exploring potential energy-saving measures within the healthcare sector. These include reducing scanner energy consumption through improving workflow efficiency, optimizing cooling systems, and exploring energy conservation technologies [9]. Our findings underline the power of protocol optimization, with significant energy and time reductions achieved simply by refining the protocol settings. For example, in knee imaging, this strategy (economic protocols) alone resulted in a 9% reduction in energy consumption and a 24% reduction in scan time. Such savings are crucial as they demonstrate the potential for efficiency improvement without the need for any additional technology or financial investment. However, we observed even greater energy savings when DL technologies were employed. In knee imaging, this advanced method allowed for an energy consumption reduction of 89% and a time reduction of 66% compared to the baseline. The strong correlation between the reduction in scan time and energy consumption suggests the increased computational demand for DL technologies is offset by the shorter acquisition times. In a scenario encompassing 250 working days per year, with daily operation times ranging from 8 h to 12 h, significant financial savings can be realized through these methodologies. Utilizing the economic power mode results in annual savings of 4881 € ($5287) per scanner. Even greater savings are achievable if DL protocols are implemented, leading to an additional reduction of 7032 € ($7617) or 5422 kg CO_2_ per scanner per annum. Even the to-be-expected increased computational demand of DL methods (and, by that, increased energy consumption for the image reconstruction) was compensated by the energy reduction during scanning for these shorter acquisition times. This highlights the potential efficiencies and cost-reduction strategies that may be gained through the thoughtful application of these technologies.

We acknowledge several limitations of this study. First, not all energy reduction options were available at all scanners to provide an on-site comparison of the respective effects and the investigated solutions are vendor-specific. However, relative energy savings were reproducible at different sites, so we do not expect any biased influence on the results. Future studies will investigate on-site and on-scanner comparisons between the different energy-saving methods. Furthermore, future investigations will target more body regions and a larger scanner fleet in a hospital and practice setting to capture more diversity in routine examinations. Second, measurements were only performed on a 10-min interval, resulting in averaged energy consumption readings and linear interpolations to the actual start or end times of the imaging sequence. While this can have an effect on the fine-grained analysis of energy consumption, the overall global assessment is only minor impaired. From a statistical point of view, over the course of four months, these interpolation offsets are negligible. Third, the analysis was not performed on individual short protocol steps but on completed examinations that are of a comparable duration. Fourth, it is essential to note that energy costs can vary significantly between different countries and even within countries over time. This variation could affect the generalizability of our cost-saving findings, as energy savings in one region might not translate to similar financial savings in another due to differing energy prices. Fifth, our study’s findings are primarily applicable to outpatient radiology practices. In scenarios where MRI devices are operated continuously (24/7), such as in inpatient settings, the benefit would primarily stem from using DL accelerated sequence acquisition, as the opportunity for energy savings through device shutdowns or reduced operation times at night is unavailable. This distinction in the study’s setting could influence the applicability of our results in different clinical environments. Sixth, although energy consumption during the manufacturing of an MR scanner outweighs the annual operation usage, we believe that the highlighted benefits of this study (shortened scan times or extended operational hours to increase the number of scans per scanner) are motivating and will contribute to a larger conversation about efficiency and sustainability in medical imaging.

## Conclusion

Our findings suggest economic protocols, DL accelerated sequence acquisition, and optimized magnet cooling for effective improvement of energy efficiency in radiology departments and practices. Adopting these strategies can optimize energy consumption, reduce costs, and mitigate the healthcare-related environmental footprint. Our research provides a roadmap for similar future studies in other high-energy-consuming devices within the healthcare sector. For a more in-depth temporal analysis, future studies will be directed towards a higher time-resolved energy consumption measurement, which will shed further light on the energy consumption behavior of individual scan steps and different parameter settings within these steps.

## Abbreviations

CT: Computed tomography
DL: Deep learning
EPM: Eco power mode
MRI: Magnetic resonance imaging
